# Circulating tumor cells: biology and clinical significance

**DOI:** 10.1038/s41392-021-00817-8

**Published:** 2021-11-22

**Authors:** Danfeng Lin, Lesang Shen, Meng Luo, Kun Zhang, Jinfan Li, Qi Yang, Fangfang Zhu, Dan Zhou, Shu Zheng, Yiding Chen, Jiaojiao Zhou

**Affiliations:** 1 Department of Breast Surgery, the Second Affiliated Hospital, Zhejiang University School of Medicine, Hangzhou, China; 2 Cancer Institute (Key Laboratory of Cancer Prevention and Intervention, China National Ministry of Education), the Second Affiliated Hospital, Zhejiang University School of Medicine, Hangzhou, China; 3 Department of Breast Surgery, the First Affiliated Hospital of Wenzhou Medical University, Wenzhou, China; 4 Department of Pathology, the Second Affiliated Hospital, Zhejiang University School of Medicine, Hangzhou, China; 5 Department of Surgery, Traditional Chinese Medical Hospital of Zhuji, Shaoxing, China

**Keywords:** Tumour biomarkers, Tumour biomarkers

## Abstract

Circulating tumor cells (CTCs) are tumor cells that have sloughed off the primary tumor and extravasate into and circulate in the blood. Understanding of the metastatic cascade of CTCs has tremendous potential for the identification of targets against cancer metastasis. Detecting these very rare CTCs among the massive blood cells is challenging. However, emerging technologies for CTCs detection have profoundly contributed to deepening investigation into the biology of CTCs and have facilitated their clinical application. Current technologies for the detection of CTCs are summarized herein, together with their advantages and disadvantages. The detection of CTCs is usually dependent on molecular markers, with the epithelial cell adhesion molecule being the most widely used, although molecular markers vary between different types of cancer. Properties associated with epithelial-to-mesenchymal transition and stemness have been identified in CTCs, indicating their increased metastatic capacity. Only a small proportion of CTCs can survive and eventually initiate metastases, suggesting that an interaction and modulation between CTCs and the hostile blood microenvironment is essential for CTC metastasis. Single-cell sequencing of CTCs has been extensively investigated, and has enabled researchers to reveal the genome and transcriptome of CTCs. Herein, we also review the clinical applications of CTCs, especially for monitoring response to cancer treatment and in evaluating prognosis. Hence, CTCs have and will continue to contribute to providing significant insights into metastatic processes and will open new avenues for useful clinical applications.

## Introduction

**Fig. 1:**
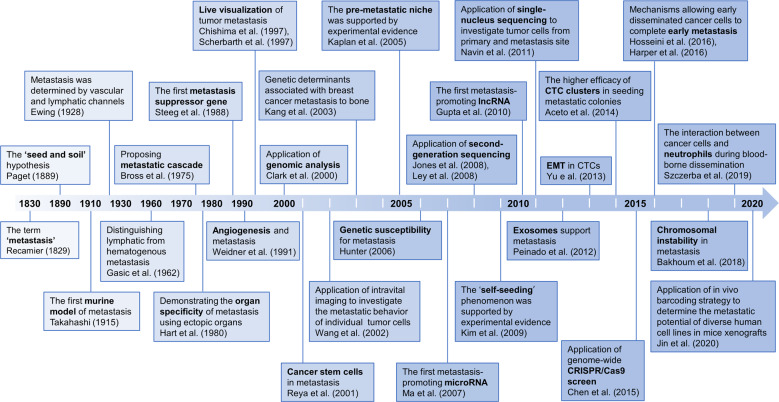
The historical milestones of cancer metastasis research

Metastasis is the most lethal feature of cancer^1^. Despite significant developments in cancer diagnosis and treatment over the past centuries, metastasis remains a major obstacle to improving clinical outcomes of cancer patients^2^. Nevertheless, we have witnessed significant progress over the past two hundred years in revealing fundamental concepts underlying the development of metastasis and in creating new technologies to facilitate cancer metastasis research. Fig 1 highlights the key discoveries and milestones in the study of cancer metastasis. The ‘seed and soil’ hypothesis by Steven Paget^3^ in the 1830s vividly clarified the progress of cancer metastasis. With the advances of science and technologies, particularly since the 2000s, a plethora of new technologies have been advanced, such as high-throughput sequencing^4,5^, transgenic mouse models^6^, CRISPER/Cas9 editing tools^7^, and single-cell sequencing^8^. With these powerful technologies, the biological phenomena underlying metastasis, such as epithelial-mesenchymal transition (EMT) of tumor cells^9^, the role of exosomes in supporting metastasis^10^, circulating tumor cells (CTCs) and CTC clusters in seeding metastatic colonies^11^, and complex interactions between tumor cells and microenvironment^12^, have gradually been unmasked, along with the discovery of numerous metastasis-related driver genes. The ‘black box’ of metastasis is gradually being unveiled, and effective metastasis-targeting agents are believed to be on the horizon in the near future.

**Fig. 2:**
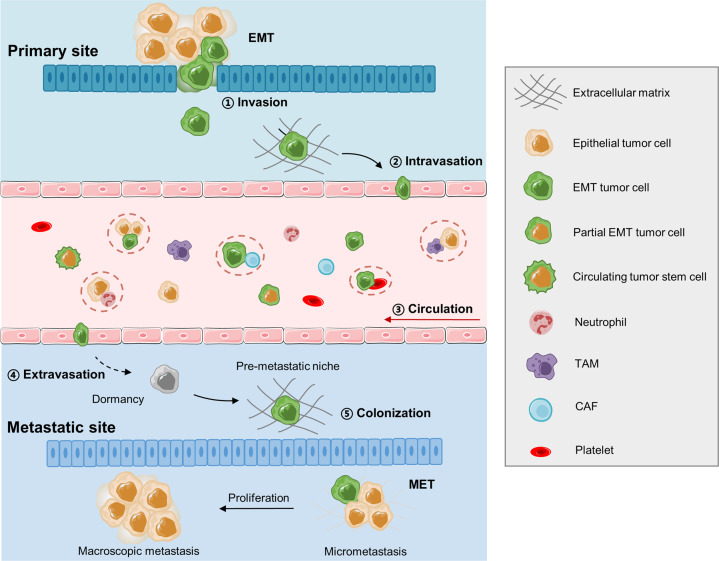
Multistep process of cancer metastasis. The complex metastatic process includes tumor cell invasion in the primary site, intravasation into circulation, survival in the circulation as CTCs and interaction with blood cells, extravasation from the circulation, attachment to and colonization of the metastatic site. EMT: epithelial to mesenchymal transition, CAF: cancer-associated fibroblast, TAM: tumor-associated macrophage

Cancer metastasis is a complex multistep process involving cancer cell invasion in the primary site, intravasation into circulation, survival in the circulation, extravasation from the circulation, and attachment to and colonization of the metastatic site (Fig. 2). CTCs are defined as tumor cells that have been sloughed from the primary tumor and are swept away by the circulatory or lymphatic systems. To date, most CTC research has focused on CTCs in the blood circulation. CTCs were first described in 1869 by Ashworth who observed “some cells” in the blood of a metastatic cancer patient with an appearance similar to tumor cells in the primary tumors^13^. CTCs have been assumed to be the substrate of metastasis. Although CTCs originate from the primary tumor, they are distinct from primary tumor cells^14^, with EMT transition properties that help them break free from the primary tumor and facilitate intravasation into the bloodstream, dissemination in clusters of CTCs to increase metastatic potential, and exhibit stemness features that enhance their ability to initiate metastasis (Fig. 2). However, most CTCs perish in the circulation, and only limited CTCs survive and infiltrate distant organs. Interactions between CTCs and the blood environment (Fig. 2), including how CTCs escape immune surveillance in the blood, have been widely implicated in the metastatic mechanisms of CTCs. It has taken more than a century for researchers to recognize the critical role of CTCs in cancer metastasis, due to the unique technical challenges required to isolate these very rare CTCs from the massive pool of circulating blood cells^15^. However, in the past two decades, emerging technologies for CTC isolation have allowed research on the biology of CTCs and have facilitated the clinical applications of CTCs in cancer screening, treatment response monitoring, and prognosis evaluation.

In this review, the biology of CTCs, as well as their interaction with the blood microenvironment, is fully reviewed. In addition, the growing number of highly sophisticated CTC enrichment and isolation technologies will be summarized. Finally, we also discuss the tremendous potential of CTCs in clinical applications.

## The biology of CTCs

### Molecular characterization of CTCs

Experimental evidence has supported the notion that tumor cells can spread even during the early stages of tumor evolution^16,17^. The molecular characterization of CTCs will add knowledge to the underlying mechanism of metastatic processes, contributing to early diagnosis and prevention of metastasis.

#### Molecular markers of CTCs

A panel of molecular markers has been used to detect CTCs in various cancers. CTC-associated markers used for different cancers are summarized in Table 1^18,19^. As most cancers are of epithelial origin, the most common marker used for CTCs is EpCAM, a “universal” epithelial marker of cancers^20^. EpCAM expression varies among different cancer types^21^, and EpCAM-based CTC detection technologies are widely applied for cancers that strongly express EpCAM, such as breast and prostate cancer. Many studies have shown that CTCs in breast and prostate cancer are EpCAM-positive, and have validated their prognostic value in either early or metastatic stage cases^22,23^. Other epithelial-derived cancer types, such as pancreatic^24^, colorectal^25^, and hepatocellular cancers^26^, also have a considerable detection rate of EpCAM-positive CTCs. Similarly, the presence of these EpCAM-positive CTCs predicts early distant metastasis and poorer survival of patients^25,27,28^. However, using EpCAM as a CTC marker has limitations. It cannot be used in tumors that are EpCAM-negative or with low expression, such as neurogenic cancers. CTCs can undergo EMT, and epithelial markers, including EpCAM, are down-regulated during EMT, which affects the detection rate of EpCAM-positive CTCs. Although there are doubts as to whether EpCAM-based technologies are appropriate to detect all CTCs, numerous studies have illustrated the potential value of EpCAM-positive CTCs in clinical applications^29^. To some extent, EpCAM-positive CTCs are a substantial subgroup of all CTCs, thus EpCAM-positive CTCs still could be a reliable biomarker if the cancer prognosis and therapeutic efficacy is relevant to EpCAM-positive CTCs.

**Table 1:** Molecular markers used to identify CTCs

Cancer types	Epithelial markers	Mesenchymal markers	Specific markers
Breast cancer	EpCAM/CK8,18,19^234,237,265–280^	Vimentin^280–283^	HER2^37–46^
	CK 5/7/8/18/19^9^	Twist^253,282,284^	ER^39,48–50^
	E-Cadherin^9,280,281^	Fibronectin^9,280^	AR^285^
		N-Cadherin^9,280,286^	MRP^48^
		SERPINE1/PAI1^9^	
		β-catenin^281^	
Prostate cancer	EpCAM/CK8,18,19^287,288^	Vimentin^102,289–291^	PSMA^51–53^
		Twist^290,291^	PSA^239^
			EGFR^51^
			AR­V7^256,292–294^
			PIM1^295^
			AR ^v567es^^294^
Kidney cancer	EpCAM^240^	-	CD147^296^
Bladder cancer	EpCAM/CK8,18,19^297–300^	-	-
Colorectal cancer	EpCAM/CK8,18,19^301,302^	Vimentin^252,303–305^	PI3K α^306^
		Twist^252,303,305^	CEA^307–309^
		SNAI1^303,305^	PRL3^252^
		AKT2^303,305,306^	
		LOXL3^310^	
		Plastin3^311^	
Non-small-cell lung cancer	CK7/8/18/19^312,313^	Vimentin^109,313,314^	Folate receptor^54–56^
	EpCAM/CK8,18,19^109,313–319^	Twist^313^	Telomerase activity^320^
		N-Cadherin^314^	
		AXL^313^	
Small-cell lung cancer	EpCAM/CK8,18,19^79,320–326^	Vimentin^242,327^	DLL3^242^
Pancreatic cancer	EpCAM/CK8,18,19^328–332^	Vimentin^257,333–335^	-
		Twist^334^	
		KLF8^335^	
Hepatocellular carcinoma	EpCAM/CK8,18,19^336–340^	Vimentin^336,341–343^	GPC3^344,345^
	EpCAM,CK19^346^	Twist^336,341–343,347^	ASGPR^344^
	EpCAM^348^	-	-
Gastric cancer	EpCAM/CK^244,349–351^	Vimentin^352^	XAF1^353^
	CK19/CK20^354^	N-Cadherin^355^	MT1-MMP^356^
			Survivin^57^
			HER2^47^
Esophageal cancer	EpCAM/CK8,18,19^357^	-	-
Cervical cancer	EpCAM/CK8,18,19^358^	-	-
Melanoma	-		MART-1^62,63^
			HMW-MAA^59,61,65,359^
			CD146^61,65,359^
			MAGE A3^62,63,66^
			GalNAc-T^63^
			MAGE A1-6^360^
			hTERT^360^
			MLANA^66^
			B4GALNT1^66^
			PAX3^62,66^
			DCT^66^

CTC markers mainly includes the epithelial markers, the mesenchymal markers, and the cancer specific CTC markers

*ARV7* androgen-receptor splice variant 7, *ASGPR* asialoglycoprotein receptor, *CEA* carcinoembryonic antigen, *EGFR* epidermal growth factor receptor, *GPC3* glypican 3, *MAGE A1-6* melanoma antigen-encoding gene family member A1-family) member A6, *MRP* multidrug-resistance-related proteins, *PI3K α* phosphatidylinositol 3-kinase α, *PIM1* proviral integration site for the Moloney murine leukemia virus-1PRL3 phosphatase of regenerating liver-3, *PSA* prostate specific antigen, *PSMA* prostate-specific membrane antigen, *SERPINE1/PAI1* serpin peptidase inhibitor, clade E, *XAF1* XIAP-associated factor 1

Due to the EMT activity of some epithelial cancer cells, detecting only EpCAM-positive CTCs probably underestimates the actual total CTC population and misses important biological information of EpCAM-negative CTCs. In some cancer types, such as non-small-cell lung cancer (NSCLC) patients, it was even found that the quantity of EpCAM-negative CTCs was significantly larger than EpCAM-positive CTCs^30^. Nevertheless, the poor isolation of CTCs by EpCAM-based technologies can be rescued by using both epithelial and mesenchymal cancer markers, as well as by marker-independent detection methods. For example, in breast cancer, the use of fluorescent-magnetic nanoparticles consisting of a dual-antibody interface targeting both EpCAM and N-cadherin has contributed to high-efficiency isolation and rapid identification of CTCs^31,32^. In biliary tract cancer, a single-cell assay for detecting CTCs allowed identification of both epithelial CTCs and non-conventional CTCs which lacked epithelial and leukocyte markers, and therefore led to an increase of CTC positivity rate^33^. The EMT program of cancer cells exhibits molecular alterations, including decreased expression of epithelial markers (E-cadherin, ZO-1, claudins, and occludins) and increased expression of mesenchymal markers (vimentin, N-cadherin, fibroblast-specific protein1, and fibronectin)^34^. EMT is executed by EMT-related transcription factors, mainly belonging to the SNAIL, TWIST, and ZEB families^34^. All these EMT-related molecules can theoretically be used for EMT-CTCs targeting methods. However, many EMT-related molecules are cytoplasmic or nuclear proteins, precluding their usage in the currently available membrane molecular-based technologies of CTC detection. Proteins such as E-cadherin, vimentin, and twist were most often used in the past^35^(Table 1), probably because of their accessibility of detection in traditional CTC detection technologies including flow cytometry sorting, immunostaining, and fluorescence in situ hybridization (FISH) staining. However, the emergence of single-cell CTC sequencing technologies^36^ will make it possible to unmask the EMT status of CTCs more comprehensively, and can cover all the EMT-related molecular alternations at the RNA level.

Other biomarkers, such as human epidermal growth factor receptor-2 (HER2)^37–47^, estrogen receptor^39,48–50^, prostate-specific membrane antigen^51–53^, folate receptor^54–56^, and survivin^57^, have been described as CTCs markers in different cancers, with different clinical significance. These cancer-specific CTC markers are listed in Table 1. Most of these cancer-specific CTC markers are in accordance with the specific molecular markers of the primary tumor. However, there is discordance in the expression of specific markers between the primary tumor and CTCs. For example, the rates of discordance of HER2 gene amplification between CTCs and primary breast tumor were around 15%^58^, suggesting a clonal selection of CTCs or clonal acquisition, probably due to genetic instability. It should be mentioned that for melanoma, a skin cancer that begins in melanocytes, the detection technologies of CTCs are based on several melanoma cell adhesion molecules, such as HMW-MAA^59–61^, MART-1^62–64^, CD146^61,65^, and MAGE A3^62,63,66^, which are very specific molecular markers for melanoma.

The variety of CTCs markers indicates the heterogeneity of CTCs among different cancer types. Even in one patient, CTCs are spatio-temporally heterogenous, which may be the result of a spatially different microenvironment in the blood and temporal changes in therapy response. Thus, it is difficult to define the entire CTC population using the very limited molecular markers currently available. In addition, CTC markers should not be constant among different stages of cancer and treatment periods.

#### Genome analysis of CTCs

Genomic instability contributes to tumor evolution and the emergence of resistant tumor subclones. Monitoring tumor genomic instability, especially in terms of tumor resistance and metastases, greatly contributes to the evaluation of treatment response and precision medicine. The evaluation of CTCs assessment using noninvasive liquid biopsy is accessible for serial sampling to detect the genomic instability of the tumor.

Determining the status of EGFR and KRAS mutations is crucial for guiding treatment in NSCLC patients receiving EGFR tyrosine kinase inhibitors and colorectal cancer patients treated with anti-EGFR therapy respectively. The concordance of mutations between CTCs and matched primary or metastatic tumor tissue has attracted much attention. Using a microfluidic technique to capture CTC, Maheswaran et al. found that only two of 31 patients with mutations were overlooked from their detection assay^67^. They identified the EGFR activating mutation in CTCs in 92% of metastatic patients with NSCLC and detected the drug-resistant mutation T790M in CTCs of 33% of patients who responded to tyrosine kinase inhibitor therapy and in 64% of patients who exhibited clinical progression^67^. For the analysis of the KRAS gene mutation, the mutational concordance rate between CTCs and matched primary tumors ranged from 37% to 90% in colorectal cancer cases^68–71^. This difference in the concordance rate may be due to the different CTC selection protocols used in these studies. KRAS mutations are also common in pancreatic ductal adenocarcinoma (PDAC), present in 90% of PDAC cases. However, Kulemann et al. found that the discordance rate of KRAS mutation in CTCs and corresponding PDAC tumors was 42%^72^. Studies of gene mutation analysis in CTCs were also conducted in many cancers such as prostate cancer^73,74^, breast cancer^75,76^, hepatocellular carcinomas^77^, and a mutational discordance between CTCs and corresponding tumors were often found. The discordance rate was probably attributed to the different detection efficiency of CTC mutations, or the heterogeneity between CTCs and primary tumor cells. Genomic assessment of tumor tissue and CTCs can be complementary. So, a combination of mutational testing of CTCs and tumor specimens would guide treatment more precisely.

Determining copy number alternations (CNA) of CTCs helps analyze and track cancer profiles as tumors evolve. In lung cancer, Ni et al.^78^ found that CTCs exhibit reproducible CNAs patterns, similar to those of metastatic tumors, and different patients shared similar CNAs patterns. In small-cell lung cancer, a CNA-based classifier for CTCs correctly assigned 83.3% of patients as chemorefractory or chemosensitive^79^. In breast cancer, the assessment of CNAs in archived CTCs is feasible. Paoletti et al.^80^ found that the CNAs of CTCs and paired metastatic tumor tissue in breast cancer patients were highly concordant, although CTCs and matched tumor tissue harbored several discordant copy number alterations, suggesting that CTCs were the subclone cells of tumor tissues. In triple-negative breast cancer, CTCs with chromosome 10 and 21q CNAs are predictive of clinical progression, and their network analysis presented connected modules including HER/phosphatidylinositol-4,5-bisphosphate 3-kinase/RAS/JAK signaling^81^. In prostate cancer, Lambro et al.^82^ revealed that CNAs of CTCs were interpatient and intercell heterogeneous, and could be missed in bulk biopsy analyses. In metastatic castration-resistant prostate cancer, whole genomic copy number analysis of CTCs showed that common genomic gains in CTCs involved genes such as androgen receptor (AR), mesenchymal-to-epithelial transition (MET), ERG, and cyclin-dependent kinase 12, while common genomic losses were observed in genes such as phosphatase and tensin homolog (PTEN), RAF1, and GATA2^83^. Similarly, Malihi et al. also observed that CNAs in genes including PTEN, RB1, TP53, and AR closely associated with genomic instability and survival in aggressive variant prostate cancer^84^.

Other genome analyses have also been conducted in CTCs. FISH testing was adopted in CTCs to detect biomarkers for treatment sensitivity, such as ALK FISH testing in CTCs of NSCLC patients^85^ and HER2 FISH testing in CTCs of breast cancer patients^86,87^. Recently, based on the technique of single-cell resolution DNA methylation analysis, the DNA methylome of single CTCs and CTC clusters was revealed for breast cancer patients, and indicated that the CTC cluster hypomethylation profile obtained was associated with a poor prognosis and that treatment with Na+/K+-ATPase inhibitors to dissociate CTC clusters could revert the methylation profile of CTC clusters and suppress metastasis^88^.

#### Transcriptome analysis of CTCs

Single-cell sequencing has developed rapidly in recent years and has been applied to investigate the CTC transcriptomes. Single-cell expression profiles can distinguish CTCs from mesothelial cells and blood cells in lung adenocarcinoma, with representative markers including EpCAM for CTCs^89^. Single-cell sequencing-based transcriptome analysis revealed heterogeneity in the CTC subpopulation. By testing the expression of proliferation-associated genes such as the Ki-67 proliferation marker, Magbanua et al^90^. found that 65% of CTC in patients with metastatic breast cancer had low proliferation Ki-67 and that the 35% of patients with a high proliferation Ki-67 expression had a poor prognosis. Cheng et al.^91^ performed a single cell transcriptome analysis of 666 CTCs in patients with metastatic breast cancer. They determined that intra-patient CTCs were heterogeneous with regard to EMT-like and MET-like states, and CTCs were enriched for the stem-like phenotype^91^. Single-cell sequencing of CTCs also greatly helped in discovering driver signaling pathways that contributed to metastasis and treatment failure. RNA-Seq of single prostate CTC indicated the activation of noncanonical Wnt signaling in antiandrogen resistant patients^92^. Further, using mouse models, ectopic expression of Wnt5a attenuated the effects of an AR inhibitor and suppression of Wnt5a could partially restore the sensitivity in drug-resistant prostate cancer cells^92^. CTCs have been associated with a poor prognosis in colorectal cancer. A study of the CTC-specific transcriptome profile^93^ of six metastatic colorectal cancer patients characterized 410 CTC-specific genes, which were primarily related to cell movement and adhesion, such as VCL ITGB5, bone morphogenetic protein 6, transforming growth factor beta 1, and talin 1, and were related to cell death and proliferation, such as amyloid beta precursor protein, clusterin, and TIMP1.

### Epithelial-to-mesenchymal transition of CTCs

EMT is the process by which epithelial tumor cells lose their intercellular adhesion and acquire mesenchymal and invasive properties. During dissemination, tumor cells detach themselves from the basement membrane through EMT activation and directly enter the circulation, serving as CTCs traveling to distant sites. When CTCs extravasate, they then undergo a reverse process termed MET and proliferate to form macro-metastases^94–96^. Herein, metastatic development depends on the delicate balance of the transition between these two phenotypes. The activity of EMT-MET was also proposed to play an important role in the metastatic process of CTCs^97^. Using mouse models, it was found that epithelial-type CTCs with a restricted mesenchymal transition had the strongest lung metastases formation capacity, whereas mesenchymal-type CTCs showed limited metastatic ability^98^. In breast cancer, CTCs exhibit dynamic changes in EMT composition, and mesenchymal CTCs were found to be closely associated with cancer progression^9,98^. A plethora of studies has shown increased EMT of CTCs rather than primary tumor cells in various cancers^99–103^. In a study based on the bioinformatics analysis of seven sets of gene chips, Guan et al. showed that compared with primary tumors, the main changes in CTCs involved cell adhesion, EMT, and apoptosis^104^. In a prospective study including 39 patients with invasive breast cancer, Tashireva et al. observed a majority of heterogeneous CTC phenotypes (22 out of 24 detectable samples) exhibiting EMT plasticity^105^. Interestingly, it was found that fluid shear stress can induce the EMT of CTCs via JNK signaling in breast cancer, which further confirmed the relationship between augmented EMT of CTCs and poor patient survival^106^.

Clinically, combining the total CTC count and the proportion of mesenchymal CTCs^107^ can be used to monitor therapeutic resistance and predict prognosis in cancer patients due to the significant survival differences of this criterion^108^. For example, since the baseline presence of total CTCs in advanced NSCLC conferred poor prognosis^109^, and the presence of over five EMT-CTCs indicated progressive disease^110^. Different numbers of total CTCs and EMT CTCs were found to play an important role in determining the prognosis of breast cancer patients. Intriguingly, it was emphasized that a better understanding of EMT-CTC subtypes and their interactions with peripheral blood mononuclear cells could help design better anti-metastatic treatments^111^. As CTC EMT-positive patients with neutrophil-to-lymphocyte ratios ≥3 had an 8.6 times increased risk of disease recurrence compared with CTC EMT-negative patients with lower neutrophil levels, inflammation-based scores increased the prognostic value of CTCs in primary breast cancer^112^. Therefore, targeting the EMT pathway may prevent tumor cell spread in early-stage patients and eradicate metastatic cells in advanced stages.

### The stemness of CTCs

Many previous studies have indicated a subpopulation of aggressive CTCs with “stemness” traits in different cancers, which refer to their properties for self-renewal and tumor growth induction. In bladder cancer, studies have reported the high expression of OCT4, a crucial stemness maintenance protein^113^, in a subgroup of CTCs^114^. In breast cancer, the CD44^+^/CD24^-/low^ and aldehyde dehydrogenase 1 (ALDH1) + cell phenotypes are reported to be associated with stemness. After detecting the expression of CD44, CD24, and ALDH1 of CTCs in 30 metastatic breast cancer patients, Theodoropoulos et al. found that 35.2% of 1439 CTCs were CD44^+^/CD24^-/low^, and 17.7% of 238 CTCs were ALDH1^high^/CD24^-/low^, providing evidence of CTCs stemness^96^. A CTC-3 cell line established from the peripheral blood cells of a breast cancer patient showed more aggressive growth than the widely used MCF-7 breast cancer cell line. Gene profiling revealed higher expression of the stemness markers in the CTC-3 cell line compared to MCF-7 cells^115^. In hepatocellular carcinoma (HCC), 71.4% of HCC patients were CTC positive for the cancer stem cell marker, CD44; thus, they had a significant population of CTCs with stem properties, which could contribute to tumor cell survival and dissemination^116^. In glioblastoma, RNA-seq analysis revealed Wnt activation-induced stemness and chemoresistance in CTCs^117^. In prostate cancer, the stem cell marker CD133 was observed in the majority (> 80%) of CTCs of patients with metastatic castration-resistant prostate cancer^97^ and a stem-like subpopulation of the C-X-C motif chemokine receptor 4+/CD133+CTCs was more prevalent in EpCAM-negative CTCs than in EpCAM-positive CTCs^118^.

It is crucial to understand the mechanisms regulating CTCs stemness, and interfering with the CTCs subpopulation stemness properties may more efficiently suppress cancer progression and relapse. CTCs undergo considerable levels of fluid shear flow during their dissemination, and the fluid shear flow itself may have an impact on CTCs. Using a model of breast cancer with brain metastasis, it was suggested that hemodynamic shear flow could upregulate the stemness genes of CTCs in surviving under conditions of shear flow^119^. EMT-like transition of CTCs by downregulating ERK and GSK3β signaling could promote the conversion of CTCs into stem-like CTCs with high sphere-forming and tumor-initiating capacity^120^.

## CTCs and the blood microenvironment

**Fig. 3:**
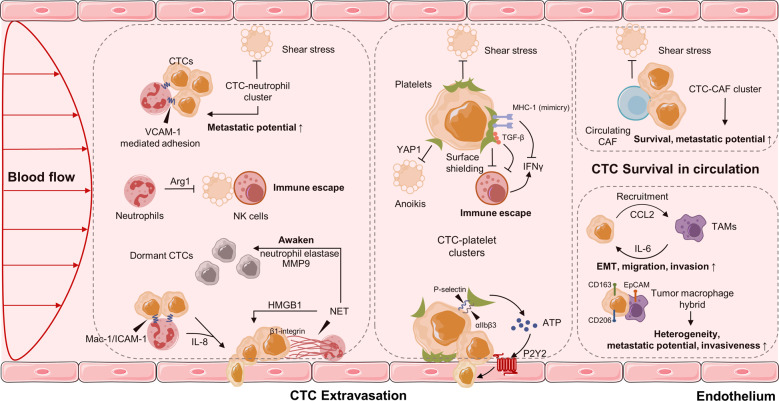
CTCs in the blood microenvironment, and their interaction with neutrophils, platelets, CAFs and TAMs. CAFs: cancer-associated fibroblasts, TAMs: tumor-associated macrophages

When transported in the bloodstream, a major of CTCs are constrained by detrimental shear stress, or die from anoikis, a programmed cell death mechanism due to loss of cell attachment^121,122^. Only a small fraction of CTCs interact tightly with platelets, neutrophils, macrophages, myeloid-derived suppressor cells (MDSCs), or cancer-associated fibroblasts (CAFs) to escape the immune system and promote their survival^123,124^. Recently, accumulating studies suggest that the interaction and modulation between CTCs and hostile blood microenvironment is essential for adhesion to endothelial cells, tissue invasion, and tumor metastasis (Fig. 3).

### Interaction of CTCs with neutrophils

Neutrophils are the most abundant circulating leukocytes in humans and have recently been studied to support cancer progression^125^. An increased number of neutrophils in circulation is associated with poor prognosis in several types of cancers^126–128^. The formation of CTC-white blood cells (WBCs) clusters was previously reported within the bloodstream^129^. In 2019, Szczerba et al. determined that CTCs were significantly associated with neutrophils in both mouse models and breast cancer patients, exhibiting more metastatic potential with greater expression of genes that involve cell cycle progression compared to CTCs alone^130^. These observations are consistent with previous findings showing the proliferation role of neutrophils on tumor cells^131^. CTC and neutrophil binding is mediated by the cell–cell junction and possibly requires the vascular cell adhesion molecule 1^130^. Furthermore, neutrophils can directly adhere to CTCs through the Mac-1/ICAM-1 interaction and act as a bridge between tumor cells and the liver parenchyma, thus promoting extravasation and liver metastasis^132^. Thus, CTCs clusters with neutrophils anchor to the vascular endothelium for extravasation while resisting shear stress, and the process is mediated by a series of cell adhesion proteins, such as cadherin, integrin, and surface glycoprotein^133–135^. Intriguingly, Chen et al. found that IL-8 secreted from neutrophils was essential for neutrophil sequestration with arrested tumor cells and for the extravasation behaviors of adjacent tumor cells through the endothelial barrier^136^.

Neutrophils can also promote metastasis in an indirect manner. Neutrophil extracellular traps (NETs) are web-like structures formed by DNA–histone complexes and proteins released from activated neutrophils, with the ability to impact on CTC biology^137^. Many studies have found that NETs were able to capture CTCs in the circulation and, in doing so, promoted metastatic dissemination^138,139^. In vitro and in vivo experiments have shown that CTC adhesion to NET is mediated by β1-integrin expressed on both NET and cancer cells, while this effect was abrogated following DNAse I administration^139^. In a murine model of surgical stress, the NET formation triggered the release of high-mobility group box 1, which activated TLR9-mediated pathways in CTCs and therefore accelerated the progression of liver metastases^140^. In addition, NETs can also awaken dormant cancer cells and promote metastasis. Recently, Albrengues et al. elegantly demonstrated that two NET-associated proteases, neutrophil elastase and matrix metalloproteinase 9 (MMP9), concentrate at laminin, provoke its cleavage, by generating an epitope that induced awakening of dormant cancer cells by integrin activation and FAK/ERK/MLCK/YAP signaling^141^. In turn, tumor-expressed protein, such as protease cathepsin, has been shown to support lung metastasis of breast cancer by promoting NET formation in metastatic niches^142^. Coiled-coil domain containing protein 25, another protein expressed on the cancer cell membrane, could serve as a specific sensor for the DNA component NETs, which induces migration and adhesion of tumor cells^143^. In addition, by forming NETs, circulating neutrophils can help CTCs escape immune surveillance by suppressing the activation of peripheral leukocytes^144^, the function of natural killer (NK) cells^145^, the antitumor response of effector T cells^146^, and even cooperation with other immune cells (such as IL17-producing γδT cells)^147^. Overall, there is evidence for a pro-metastatic role of neutrophils in their interaction with CTCs, but the specific mechanism remains to be elucidated in more detail.

### Interaction of CTCs with macrophages

Tumor-associated macrophages (TAMs) not only contribute to metastatic progression within the primary tumor, but also promote later stages of metastasis including dissemination and extravasation of CTCs^148^. Hamilton et al. tried to investigate the CTC-macrophage interactions by co-culturing peripheral blood mononuclear cells with CTC cell lines obtained from small-cell lung cancer patients. They found that CTCs were able to induce monocyte differentiation to TAMs, which secrete a host of mediators such as osteopontin, MMP9, chitinase-3-like-1, and platelet factor to promote further leukocyte recruitment, migration, and invasion^149,150^. In another study of colorectal cancer, the feedback loop between TAMs and cancer cells is essential for the EMT program of CTCs and intravasation into the blood stream. Mechanistically, IL6 derived from TAMs regulates invasiveness through the STAT3/miR-506-3p/FoxQ1 pathway, which in turn increases CCL2 expression of TAM-educated tumor cells to help recruit macrophages^151^. TAMs seem to promote CTCs acquisition of mechanical adhesiveness and endurance, thereby helping them to form protective cell clusters and confer resistance to shear stress^152^. The fusion of macrophages and tumor cells could be a potential mechanism of immune evasion and invasion. These macrophage-tumor cell hybrids were shown to have M2-like macrophage phenotypes (CD163) as well as epithelial markers (EpCAM)^153,154^, and have been isolated from the blood of patients with multiple cancers such as PDAC^155^, melanoma^154^, breast, ovarian, and colorectal cancer^156^. Furthermore, when transplanted into mice, they spread widely and formed lesions in distant tissues^154,155^. A recent study by Gast et al. revealed that fusion hybrids can increase tumor heterogeneity and metastatic behavior, further correlating with disease stage and overall survival among several cancers^157^. Furthermore, larger hybrid sizes were also associated with worse survival among patients with non-small cell lung cancer^158^. Understanding the mechanism of direct interaction and molecular fusion between CTCs and macrophages is of great significance for identifying therapeutic targets.

### Interaction of CTCs with platelets

The metastasis and progression of cancer are greatly influenced by the recruitment and activation of platelets, which support the survival of CTCs as well as their seeding and outgrowth at secondary sites^159,160^. Platelets can bind to and form aggregates with CTCs in the blood stream, and CTCs expand the formation of aggregation by releasing prothrombotic and procoagulant microparticles or by expressing tissue factor^161,162^. Platelet-released mediators, such as TGF-β, have been found to accelerate EMT in CTCs and to promote invasion and metastasis^163,164^. Xiong et al. recently determined that the expression of the heat shock protein 47 was induced during EMT, which enhanced the cancer cell–platelet interaction through its dependent collagen secretion in breast cancer cells^165^. Interestingly, platelets are believed to protect CTCs against mechanical stress^166^ and induce resistance to anoikis, the latter being mediated by the activation of the YAP1 pathway^167^. Furthermore, platelets promote escape of CTCs from NK cell attack through various mechanisms including (1) platelet aggregates that produce surface shielding to defend the cytolysis effect of NK cells;^168^ (2) platelet-derived normal MHC-I that are transferred to the surface of the tumor cell, thus preventing the identification of NK cells;^169^ (3) downregulation of natural killer group 2, member D (NKG2D) in NK cells by platelet-derived TGF-β, as well as platelet-mediated shedding of NKG2D ligands, which contribute to impaired antitumor cytotoxicity;^170,171^ and (4) platelet-derived glucocorticoid-induced TNF-related ligand which activates GITR in NK cells and reduces their cytotoxicity^172^. Furthermore, NK cells and platelets can also interfere with neutrophils, T cells, and macrophages and modulate their immune function^173–175^. In addition to safeguard CTCs within the bloodstream, platelets are also involved in the adhesion of endothelial cells. The attachment of platelets and CTCs is mediated by platelet adhesion receptors, such as the integrin αIIbβ3 and P-selectin, thereby supporting the firm adherence of CTCs to the endothelial wall^176–178^. Furthermore, tumor cell-activated platelets release ATP from dense granules, which then induces the activation of the endothelial P2Y2 receptor and allows transendothelial migration of tumor cells by increasing permeability^179^. One study has also revealed that the interplay between integrin α6β1 on platelets and its receptor, a disintegrin, and metalloprotease 9 on CTCs is necessary for the extravasation process of cancer cells^180^. Platelets could also increase vascular permeability to help tumor cell extravasation. For example, a preclinical lung metastasis model showed that tumor cell-associated CD97, an G protein-coupled receptor, can initiate platelet activation thereby leading to granule secretion, including both ATP and lysophosphatidic acid release^181^. Similarly, the interplay between platelet-specific receptor glycoprotein VI and its ligand galectin-3 expressed on colon and breast cancer cells was revealed to promote platelet activation and ATP secretion^182^. Consequently, these platelet secretions favor a process of tumor metastasis by regulating vascular permeability. Recently, Xu et al. discovered that Ptx@AlbSNO can block tumor-specific platelet functions to suppress tumor EMT as well as prevent platelet adhesion around CTCs. Ptx@AlbSNO can also inhibit TGF-β secretion and enhance intratumoral immune cell infiltration to reverse the immunosuppressive TME, thereby suppressing distant metastasis^183^. Taken together, the close and complex crosstalk between CTCs and platelets might involve distinct molecule variants and signaling pathways and possibly represent a promising antitumor strategy, particularly attractive for the treatment of several cancers.

### Interaction of CTCs with MDSCs

MDSCs are a heterogeneous subset of myeloid cells characterized by immunosuppressive properties that also promote metastatic dissemination. Under a standard protocol for isolating human MDSCs, Cassetta et al. found that polymorphonuclear (PMN)-MDSC were significantly expanded among most cancer types except melanoma compared with infection and inflammation^184^. CTC-MDSC clusters are thought to evade immune surveillance of the T cell response^185^. Indeed, a decrease in circulating MDSCs was associated with an increase in activated OX40+PD-1- T cells in patients with diffuse large B-cell lymphoma^186^. Furthermore, Sprouse et al. found that in vitro co-culture of CTCs derived from melanoma and breast cancer patients and PMN-MDSCs enhanced Notch activation in CTCs through direct interaction between Jagged1 (Notch1 ligands) expressed on MDSCs and the Notch1 receptor expressed on CTCs. The increased production of reactive oxygen species production of MDSCs could upregulate Notch1 receptor expression, therefore, promoting CTC proliferation^187^. The potential mechanisms underlying the interplay between CTCs and MDSCs remains to be determined.

### Interaction of CTCs with CAFs

CAFs are one of the most abundant components in the TME and play a prominent role in tumor initiation, angiogenesis, metastasis, and drug resistance^188^. Mechanistically, CAFs remodel the extracellular matrix structure, which allows tumor cells to invade through the stroma and communicate with cancer cells by secreting growth factors, chemokines, and cytokines^189^. However, little is known about the interplay between CAFs and CTCs. Duda et al. first demonstrated that CTCs could carry CAFs from the primary tumor to the metastatic site in mouse models of lung cancer metastasis^190^. These host-derived CAFs directly enhance tumor cell survival and promote the formation of metastasis, while depleting CAFs from lungs significantly reduces the number of macroscopic metastasis and extends survival rate in mice. Moreover, CAFs can protect CTCs from the fluid shear forces during the dissemination process^191^. In a three-dimensional co-culture model, CAFs were found to induce shear resistance to prostate tumor cells through stable intercellular contact, as well as soluble factors (such as CXCL5, CCL2, and CCL7), which are associated with cell survival, invasion, and EMT. In addition to experimental models, circulating CAFs (identified by FAP and α-SMA co-expression) have been detected in the peripheral blood of patients with metastatic breast, cancer but not in patients in the early stages^192^, and exhibit excellent precision in metastatic diagnosis (AUC–ROC, 0.975) when isolated using a novel acoustic microstreaming platform^193^.

## Technologies for CTCs enrichment and isolation

Over the past few years, many methods have been proposed to capture CTCs. Due to the extremely small proportion of CTCs in patients’ blood, it is still a great challenge to accurately isolate CTCs from the numerous blood cells, and especially to invent applicable methods that can efficiently detect viable CTCs for subsequent in-depth analysis. Here, we will discuss the development of CTC-related technologies over the last two decades, as they have experienced tremendous growth. We will emphasize the most innovative methods associated with nanoscale materials or novel microfluidic chips, hoping to provide a useful framework of CTC-related technologies.

Generally, there are three core strategies of CTCs technologies^194^, which include (1) capture and enrichment, (2) detection and identification, and (3) release. The first strategy of capture and enrichment involves a specific interaction between CTCs and materials through physical interactions or antibody–antigen interactions. The second strategy of detection, which means identifying the CTCs, refers to various methods, such as fluorescence microscopy, fluorescence spectrophotometry, flow cytometry, surface-enhanced Raman scattering, or electrical impedance. In the last strategy, released CTCs are mainly used for downstream analysis, such as genomics, transcriptomics, proteomics, and CTCs culture.

### Classic CTC-related technologies based on physical properties

The physical separation enrichment method of CTCs is based on differences between CTCs and blood cells in size, density, deformability, and electrical properties. The Isolation by size of epithelial tumor cells system^195^ can filter blood samples through an 8-μm diameter polycarbonate TRACK-ETCH-type membrane, but it has low efficiency. An improved method, which consists of a pressure regulating system, the flexible micro spring array device^196^, reaches a capture efficiency of 90% with a detection of CTCs in 76% of samples. However, there are various trends of CTC counts observed from different samples, making this method not reliable for widespread use. CTCs can also be sorted using the Oncoquick system^197^, a density-dependent technique that allows red blood cells and WBCs to be filtered, or by Apostream^198^ that uses dielectric electrophoresis techniques in the microfluidic chamber to capture CTCs. These systems require large volume of blood and cannot collect the CTCs of a similar size as WBCs, which are their main limitations. Overall, the methods based on physical properties are generally inefficient, poor in purity, and lack of specificity, although the vitality is good and the cost is relatively inexpensive.

### Classic CTC-related technologies based on biological properties

Biological property-based technology is another important method for CTC detection. Based on antibody–antigen interaction, CTCs are usually positively enriched using epithelial (EpCAM) and mesenchymal (vimentin) markers as well as negatively enriched by using CD45 to deplete unwanted leukocytes^199^. EpCAM-dependent techniques are most commonly used by researchers. The CellSearch system^200^, the only FDA-approved device for clinical use, employs EpCAM antibody-coated ferromagnetic beads to enrich CK + /CD45-/DAPI + CTC and remove CK-/CD45 + /DAPI + WBCs. However, CTCs strongly adhere to the surface of the equipment in antibody interaction-based methods, making them difficult to be released. This deficiency can be resolved in another EpCAM-dependent, MagsWeeper system^201^, which uses a magnetic rod to enrich CTCs and eliminate cells not bound to magnetic beads, allowing the release of CTCs for the following biochemical analysis. Canpatrol^TM^ ^202^ is another representative of the EpCAM-dependent technique, which provides morphological, cytological, and genetic characterization of individual CTCs. In summary, techniques based EpCAM are extensively-used. However, because the CTC surface antigen has high heterogenicity, CTCs that have low expression of EpCAM may not be enriched, causing inaccurate results, while methods based on physical properties do not have this limitation. Therefore, combining the advantages of different technologies or looking for CTCs with high sensitivity and specific tumor markers have gradually won the attention of researchers.

Recently, with the development of microfluidic chips, nanomaterials, and next-generation sequencing, researchers have many advanced technologies to stimulate progress in CTC-related technologies. Importantly, researchers are trying to reach higher levels of CTC-related technologies in several key parameters: yield, purity, enrichment ratio, throughput, viability, sensitivity, specificity, release rate, accessibility for further analysis, and simplicity of equipment operation).

### Recent CTC-related technologies: microfluidic-based and nanotechnology-based techniques

Besides the classic CTC-related technologies discussed above, some newer technologies, such as microfluidic-based and nanotechnology-based techniques have been developed. Microfluidic-based cell sorting approaches use “intrinsic” (e.g., fluid dynamic forces) versus “extrinsic” external forces (e.g., magnetic, electric field, acoustic, and optical forces) to separate cells, and then select target cells from a sample of heterogeneous cells through different physical and biological properties^203^. The CTC-chip is a silicon microfluidic platform on which the CTCs are captured on the slides of molecular marker coated posts. The CTC-chip^204^ can separate viable CTCs from whole blood without pre-labeling or processing of samples, resulting in increased cell activity and separation purity. A modified chip-based platform using gold nanoparticles on a herringbone chip (NP-^HB^ CTC-Chip^205^) easily detaches viable CTCs and safely releases cells for further analysis by utilizing a chemical ligand-exchange reaction with gold nanoparticles on a herringbone chip. Furthermore, the monolithic CTC-iChip^206^ has high-efficiency WBC depletion and allows the characterization of CTCs with epithelial and mesenchymal characteristics. Although these microfluidic chips have greatly contributed to the development of the detection of CTCs (i.e., improved capture efficiency, viability, and depletion of WBCs), they are not widely applied for clinical use due to limitations, which include long set-up time, high initial cost, bulky instrumentation, and limited ability to perform single-cell molecular analysis.

In an attempt to capture CTCs in an automated manner (Table 2), Zhang et al. successfully sorted MCF-7 cells from a 5 mL volume of diluted blood within 23 m with a recovery rate of 85%^207^. Even more remarkable, Jia et al. developed a less costly self-driving micro-cavity array chip to achieve cell loading, lysing, isothermal amplification, and signal read-out on a single chip^208^. This novel chip can perform genetic analysis at the single-cell level, it has great potential in personalized therapy and efficacy monitoring. Furthermore, another automated and integrated microfluidic system proposed by Wang et al. is reported to achieve CTC capture and identification within 90 m. With the advantages of automation, stability, economy, and user-friendly operation, this system provides broad prospects for cancer screening and prognosis, especially in HCC^209^. Additionally, Lee et al. invented a microfluidic-based integrated system to achieve simultaneous on-chip isolation and characterization of circulating tumors utilizing differences in magnetic field gradient and immune fluorescence. Furthermore, this novel system can differentiate on-chip eight different subtypes of heterogenic CTCs, guiding the diagnosis and prognosis of breast cancer^210^. By combining the microfluidic technology and in situ molecular profiling techniques, the On-chip Post-processing Enabling chip platform has the ability to perform molecular analyses of single CTC from metastatic breast cancer and metastatic pancreatic cancer patients without any off-chip processes, suggesting its potential implementation of early molecular detection for cancer metastasis^211^. Taken together, less costly automated and integrated microfluidic systems that allow easy CTCs detection and cell analysis have great clinical value.

**Table 2:** The CTC detection technologies in recent three years (from 2018 to 2021), mainly including microfluidic chip and nanotechnology-base methods

Technology	Key features	Strengths	Weakness	Author (year)
Microfluidic chip				
Automated microfluidic method	• A fully integrated microfluidic device and a set of robust fluid-driven and control units • A flow regulatory chip and two cell separation chips	• Label-free and simple • High recovery rate (85%) • Rapid processing time (23 m)	• A need of sample dilution to avoid cell–cell interaction • Quantitative design rules are still lacking for channels	Zhang et al.^207^
Self-driving micro-cavity array chip	• Integrate sample detection structure and vacuum driving system. • Use the “film-polydimethylsiloxane chip-film” structure and oil sealing method during amplification reaction to minimize water loss.	• Less costly and simple • An excellent linear • Mutational gene profiling of single CTC • Achieve cell loading, lysing, isothermal amplification, and signal read-out on one chip.	• Air diffuse across the thin polydimethylsiloxane walls under some conditions.	Jia et al.^208^
Automated and integrated microfluidic method	• Integrate a 3D printed off-chip multisource reagent platform, a bubble retainer, and a single CTC capture microchip.	• Achieve CTC capture and identification within 90 m. • Decrease immunostaining time and antibody consumption by 90%. • Detect CTCs from various cancers.	• Deficiencies in patient recruitment	Wang et al.^209^
Integrated microfluidic method	• Use magnetic field gradient and immune-fluorescence differences.	• Isolate CTCs with an efficiency of >99%. • Differentiate eight different subtypes of heterogenic CTCs • Simultaneous on-chip isolation and discrimination of CTCs.	• Limitation of distinguishable fluorescence wavelength light of microscope	Lee et al.^210^
Nanotechnology-based methods				
Magnetic nanoparticles				
Tannic acid-functionalized magnetic nanoparticles	• Tannic acid interact with glycocalyx on cancer cells	• Inhibit the nonspecific adhesion of peripheral blood mononuclear cell. • 95.1% of capture purity for MCF-7 cells	• EpCAM-dependent	Ding et al.^214^
Peptide-Based Magnetic Nanoparticle	• Use N-Cadherin recognition peptide-functionalized magnetic nanoparticles	• High capture efficiency (about 85%) of mesenchymal CTCs from spiked human blood • Distinguished epithelial and mesenchymal subgroups.	• Unmentioned	Jia et al.^213^
CoFe2O4@Ag magnetic nanohybrids	• Gold electrodes are modified with MXene nanosheets and an HB5 aptamer is immobilized on the MXene layers • CoFe2O4@Ag magnetic nanohybrids is bonded to the HB5.	• A wide linear range of 10^2^-10^6^ cells/mL, low detection limit of 47 cells/mL • Good sensitivity and selectivity in the detection of HER2-posetive cells in blood samples. • Cost-effective and environmentally friendly	• Ag has some aggregations. • The magnetic property of magnetic probes can affect the isolation of magnetic cells.	Vajhadin et al.^216^
Gold nanoparticles				
Two-dimensional nanozyme with gold nanoparticles	• Au nanoparticles-loaded two-dimensional bimetallic PdMo nanozymes assembled with an aptamer composed of a thiol-modified EpCAM	Excellent analytical performance • A satisfactory CTCs release reaching a range of 93.7–97.4% and good cell viability	• Long preparation time of the Au@PdMo nanozymes • Complex detection procedure	Yang et al.^224^
AuNP–anchored black phosphorus nanosheets	• Use electrochemical detection • Use BP@AuNPs@aptamer as a probe combined with immunomagnetic separation	Good analytical performance with a detection limit of 2 cell mL^−1.^ • Good practicality in detecting MCF-7 cells	• EpCAM-dependent	Liu et al.^221^
Antibody-Functional Microspheres Integrated Filter Chip	• Consist of a semicircle arc and arrays • Use interfacial zinc oxide coating with nanostructure on the surface of the microsphere to increase specific surface area	• Separate large-scale CTC-microspheres. • High capture efficiency (>90%) for different tumor cell lines • Easy to find and isolate the CTCs	• Unmentioned	Wang et al.^225^

With the progress of nanomaterials, nanotechnology-based methods are becoming promising tools for CTC detection at an early-stage disease and for the monitoring of cancer development, as well as in vivo imaging^212^. Nanomaterials have a large surface-to-volume ratio and allow CTC isolation at high specificity and CTC detection at high sensitivity by adsorbing numbers of targeting ligands to bind specific molecules on cancer cells. At present, studies have reported many types of nanomaterials (Table 2) for CTC detection, including magnetic nanoparticles^213–217^, gold nanoparticles^218–220^, and quantum dots^221–223^. For example, studies have shown that the utilization of tannic acid-functionalized magnetic nanoparticles^214^, CoFe2O4@Ag magnetic nanohybrids^216^, and peptide-based magnetic nanoparticle^213^, enhances the capture efficiency of CTCs in breast cancer patients. Among them, peptide-based magnetic nanoparticles can distinguish epithelial and mesenchymal CTC subgroups and allow analysis at the single-cell level, the detection effects of which are supported by magnetic nanoparticles and microfluidic-based integrated systems^210^. Regarding gold nanoparticles, there have been significant developments. For example, the cytosensor proposed by Yang et al.^224^ showed excellent analytical performance, with a wide linear range, satisfactory CTC release (93.7–97.4%), and good cell viability. Liu et al. reported that gold nanoparticle-modified black phosphorus nanosheets improved the stability in detecting CTC^218^. Furthermore, the combination of a microfluidic system and gold nanoparticles presents a wider range of applications. Wang et al. synthesize an interfacial zinc oxide coating with a nanostructure on the microsphere surface, which increases the specific surface area and thus leads to an improved capturing efficiency of CTCs^225^. In addition, the utilization of multicolor magnetic surface-enhanced Raman scattering nanotags and chip-based immunomagnetic separation could detect four different surface protein markers on individual tumor cells in a quantitative and simultaneous manner, thus facilitating the separation of CTC subpopulations^217^.

Although nanotechnology-based techniques can provide broad prospects for CTC research in various tumors in a cost-effective and simple manner, there are limitations and challenges. First, many factors (e.g., binding of nanoparticle probes, aggregation) can affect nanoparticle-based detections, leading to decreased reliability and reproducibility. Second, most nanoparticle-based assays are prepared for academic studies, and they are still unrealistic for widely clinical translation. Third, there is possible toxicity of nanoparticles.

In the era of precision medicine, CTCs analysis has great clinical value. Tools must be sharpened first if workers are to do their job well. Therefore, CTC-related technologies are the underlying foundation to the application of CTCs in precision medicine. Herein, we reviewed previous CTCs-related technologies based on physical and biological properties, and the most recent development of techniques associated with microfluidic-based and nanoparticle-based approaches. Although there are strengths and weakness between different methods, we believe that an effective combination of these techniques may benefit CTCs research in many ways, especially the in-depth analysis and possibility in clinical applications.

## Clinical applications of CTCs

**Fig. 4:**
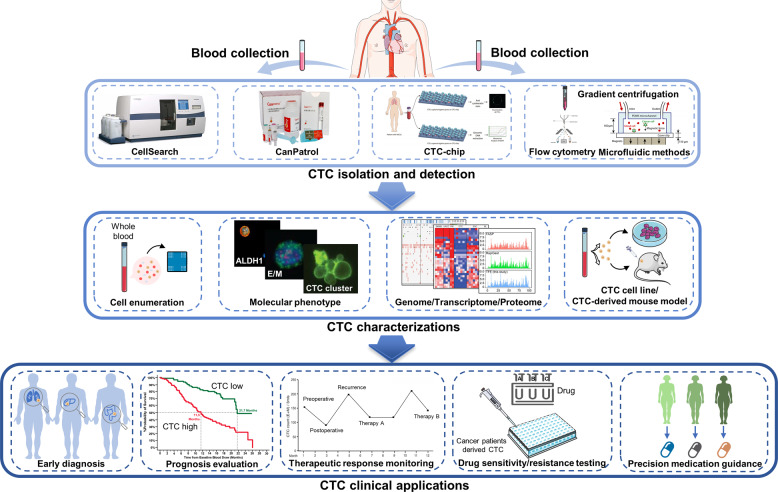
Overview of the CTC isolation and detection, characterizations, and clinical applications

Clinically, CTCs are now used as surrogate biomarkers for many solid cancers. Numerous studies have been carried out, mainly in breast cancer, prostate cancer, lung cancer, liver cancer, pancreatic cancer, gastric cancer, and melanoma. Although the clinical guidelines have not included the clinical use of CTCs, besides the inclusion of CTCs as part of the cM0 tumor classification (i.e., no clinical of overt metastasis but the detection of tumor cells in blood), many studies have predicted the great potential of CTCs in clinical applications. In this section, we will mainly present the role of CTCs as biomarkers for diagnosis, prognostication, and therapy monitoring in different cancers (Fig. 4).

### Early diagnosis of cancer

As a non-invasive method, CTC detection is attractive in assisting cancer diagnosis. Studies of CTCs used for early diagnosis of cancer in the past three years are listed in Table 3. A tumor lesion already has more than 10^9^ tumor cells by the time they are detectable in patients using current imaging procedures^15^, such as computed tomography, magnetic resonance imaging, and positron emission tomography. Diagnosis of cancers as early as possible, especially for fast-progressing cancers, is the best way to defeat them. Studies have determined that CTCs are correlated with tumor stage, but the clinical utility of CTCs in cancer detection or even in early cancer diagnosis is still a matter of debate. CTCs are considered a surrogate marker of metastatic activity, but whether metastatic dissemination of CTCs in patients occurs early during tumor formation is still controversial. However, in mouse models, early dissemination seeding metastasis has been found in breast^226,227^ and pancreatic^228,229^ carcinogenesis, indicating that CTC circulation is likely to be a very early event in cancer progression. In Barrière et al.’s study^230^, CTCs were detected in 41% of T1 stage and 47% of axillary lymph node-negative breast cancer patients, both of which are early-stage breast cancer. In the study by Thery et al.^231^ the CTC positivity rate was 21% and 24% in lymph node-negative and positive breast cancer, respectively. Based on this hypothesis, CTCs can be detected earlier before the primary tumor is visible on imaging studies, while the biggest challenge of the application of CTC application in early cancer diagnosis is indeed their scarcity and isolation. The limited sensitivity of CTC detection methods hinders their use as an effective biomarker in early cancer diagnostics.

**Table 3:** Clinical applications of CTCs in recent three years (from 2019 to 2021)

Cancer type	Author & year	CTCs utility	Detection methods	CTC marker	Patients	Main findings	Clinical Trial No.
Breast cancer	Paoletti et al.^232^	prognostic value	CellSearch	EpCAM, CK	549 mBC	In mBC patients with 1st line chemotherapy, CTC counts was associated with mortality.	NCT00382018
	Mego et al.^361^	prognostic value	RT-qPCR	TWIST, SNAIL1, SLUG, ZEB1	427 stage I-III BC	CTC EMT was detected in 18% of patients, which was associated with worse DFS.	NA
	Magbanua et al.^233^	prognostic value	CellSearch	EpCAM, CK	742 untreated BC	CTC positivity associated with reduced DRFS.	NA
	Strati et al.^253^	prognostic value	density-based isolation, RT-qPCR	EpCAM, TWIST1, CD24/CD44,ALDH1	100 early BC	Detection of TWIST1 overexpression and stem-cell transcripts in EpCAM+ CTCs provides prognostic information.	NA
	Wang et al.^255^	therapeutic monitoring	CellSearch	EpCAM, CK	160 early BC	CTC counts were lower in surgical group than trastuzumab group, but the OS rate in surgical group was higher.	NA
	Radovich et al.^279^	prognostic value	microfluidic device	EpCAM	123 early triple-negative BC	Positive CTC and circulating tumor DNA after neoadjuvant chemotherapy associated with inferior DFS and OS.	NCT02101385
	Zhang et al.^362^	recurrence monitoring	Cyttel detection	CD133, CEP8	135 early Luminal A BC	There were no differences in DFS and OS between CTC monitoring and routine re-examination monitoring group.	NA
	Magbanua et al.^234^	prognostic value, therapeutic monitoring	CellSearch	EpCAM, CK	294 ER + mBC	CTCs ≥5 was detected in 31% of the patients. Letrozole with bevacizumab gain better OS than without bevacizumab in patients with CTC ≥ 5.	NCT00601900
	Wang et al.^46^	prognostic value, guiding therapy	CellSearch	EpCAM, CK	105 HER2- advanced BC	HER2 + CTCs ≥ 2 associated with shorter survival and higher risk for disease progression (HR 2.16). Those received anti-HER2 targeted therapies had improved PFS.	NA
	Stefanovic et al.^363^	no prognostic value	CellSearch	EpCAM, CK	261 mBC	CTCs had no prognostic value in different receptor change pattern subgroups.	NA
	Papadaki et al.^254^	prognostic value	density-based isolation	CK, CD47, PD-L1	198 (100 early BC, 98 mBC)	CTCs expressing CD47 and PD-L1 have independent poor prognostic implications in mBC.	NA
	Jin et al.^364^	diagnostic value	CytoSorter	EpCAM, CK	130 BC	CTC detection rates in BC patients at Tis and T1-4 stages were 50%, 81.67%, 91.07%, 100%, and 100%, respectively.	NA
	Silveira et al.^235^	prognostic value	CellSearch	EpCAM, CK	198 HER2- mBC	CTC count ≥1 and ≥5 was detected in 37% and 22% of the patients at 4 weeks of treatment, respectively. CTCs levels at four weeks had a significant prognostic impact on PFS and OS.	NCT01745757
	Paoletti et al.^236^	prognostic value	CellSearch	EpCAM, CK	121 ER + /HER2- mBC	CTCs ≥5 at baseline was detected in 36% of patients. Elevated CTC at 1 month was associated with worse PFS.	NCT01701050
	Magbanua et al.^237^	prognostic value	CellSearch	EpCAM, CK	469 mBC	Intermediate or high CTC trajectory pattern was associated with poor prognosis.	NCT00785291
	Shliakhtunou et al.^365^	guiding therapy	magnetic isolation, RT-qPCR	EpCAM, Survivin, HER2-neu	228 BC	CTC-oriented personalized adjuvant chemotherapy (turn to taxanes or add gemcitabine) can 100% eradicate CTCs, and increase 5-year DFS by 7.4% and OS by 11.6%	NA
Prostate cancer	Sieuwert et al.^366^	prognostic value	CellSearch, RT-qPCR	EpCAM, CK, AR-Vs	118 mCRPC	CTC-adjusted detection of AR-V1 after two cycles of cabazitaxel was an independent prognostic factor for OS.	NA
	Kruijff et al.^238^	prognostic value	CellSearch	EpCAM, CK	120 mCRPC	CTC count was independently associated with PFS and OS in mCRPC patients with cabazitaxel treatment.	NA
	Graf et al.^256^	therapeutic monitoring	Streck tubes	CK, AR-V7	193 mCRPC	Patients with detectable nuclear-localized AR-V7 in CTCs had superior survival with taxanes over ARSIs.	NA
	Cie´slikowski et al.^239^	diagnostic value	CellSearch, EPISPOT, GILUPI CellCollector	EpCAM, CK, PSA, FGF2	104 PC	High CTC counts related to high-risk prostate cancer patients with occult metastases at the time of diagnosis.	NA
	Schonhoft et al.^367^	prognostic value	Epic Sciences	CK, AR	294 mCRPC	Chromosomal instability in CTCs was associated with poor OS in patients treated with AR signaling inhibitors and taxanes.	NA
	Armstrong et al.^368^	prognostic value	AdnaTest, Epic Sciences	CK, AR, AR-V7	118 mCRPC	AR-V7 in CTCs was independently associated with shorter PFS and OS with abiraterone or enzalutamide.	NA
	Xu et al.^369^	diagnostic value	Parsortix	CK, vimentin	155 PC	Combining the PSA, CTCs and the 12-gene panel, the AUC of clinically significant prostate cancer prediction was 0.927.	NA
	Sperger et al.^370^	prognostic value	VERSA	EpCAM	147 mCRPC	A transcriptional profile detectable in CTCs can serve as an independent prognostic marker in mCRPC.	NCT01942837, NCT01942837
Renal cell carcinoma	Zhang et al.^371^	no prognostic value	CanPatrol-ITMCTCs	EpCAM, CK, Beclin vimentin, TWIST,	199 RCC	No differences in the OS and DFS of RCC between the different numbers of CTCs and Beclin1 expression.	NA
	Basso et al.^240^	prognostic value	CellSearch	EpCAM, CK	195 RCC	Patients with ≥ 3 CTCs had a shorter OS.	NA
Non-small-cell lung cancer	Chemic et al.^241^	prognostic value	CellSearch	EpCAM,CK	100 NSCLC	Pulmonary venous-CTCs were detected in 48% of 100 patients, serving as early predictors of NSCLC recurrence after surgery.	NA
	Li et al.^372^	prognostic value	CytoploRare Kit, FR ligand-TaqMan	CD45, CD14	347 NSCLC	The median follow-up time was 38 months. Preoperative CTC concentration was an independent prognostic factor.	NA
Small-cell lung cancer	Messaritakis et al.^242^	prognostic value	CellSearch, Ficoll-Hypaque	Notch 1–4 receptors, CK, CD45, vimentin	108 SCLC	The detection of DLL3 + /CD45- CTCs at baseline and progression was related to decreased PFS and OS, respectively.	NA
	Wang et al.^373^	prognostic value	EpCAM-independent	EpCAM, CD45, DAPI	138 SCLC	The high number of CTC predicted adverse prognosis.	NA
Hepatocellular Carcinoma	Ha et al.^374^	prognostic value	Tapered slit filter	CK	105 HCC	Postoperative CTCs was detected in 23.8% of HCC patients and it may serve as an independent predictor of recurrence.	NA
	Chen et al.^375^	no prognostic value	Canpatrol	EpCAM, CK, vimentin, TWIST	256 HCC	CTC count and EMT status were not correlated with clinical stages or predictive of HCC recurrence.	NA
	Cheng et al.^376^	diagnostic value	CanPatrol	EpCAM, CK, vimentin, TWIST	113 HCC	Mesenchymal CTCs were increased in late-stage HCC patients. The cut-off value CTCs ≥ 1 was for the diagnosis of HCC.	NA
	Sun et al.^243^	prognostic value	CellSearch	EpCAM, CK	197 HCC	CTC count ≥3 was associated with higher risk of postoperative extrahepatic metastases.	NA
	Lei et al.^377^	prognostic value	CanPatrol	EpCAM, CK, vimentin, TWIST, Nanog	160 HCC	The numbers of EpCAM mRNA+ CTCs and Nanog mRNA+ CTCs were correlated with postoperative HCC recurrence, with Nanog > 6.7 (HR = 2.33) being the most crucial marker.	NA
Pancreatic cancer	Wei et al.^257^	diagnostic value, treatment monitoring	CytoQuest, microfluidic chip	vimentin, EpCAM, CK	100 PDAC	Vimentin+ CTCs were detected in 76% of patients with PDAC. Preoperatively higher counts was correlated with shortened RFS.	NA
	Zhao et al.^334^	prognostic value	CanPatrol	EpCAM, vimentin, TWIST	107 PDAC	CTCs were detected in 78.5% of PDAC patients. Patients with ≥ 6 total CTCs had significantly decreased OS and PFS.	NA
	Hugenschmidt et al.^331^	guiding therapy, prognostic value	CellSearch	EpCAM	209 patients	CTC-positive (≥1 CTC/7.5 mL) preoperatively showed a detrimental outcome despite successful tumor resections.	NCT01919151
Gastric cancer	Szczepanik et al.^378^	prognostic value	flow cytometry	CD45, CD44, CK	228 GC	CK + /CD44 + cells were significantly more common among patients with distant metastases.	NA
	Miki et al.^379^	prognostic value	Ficoll	EpCAM, CEA, CK	150 GC	The number of EpCAM − /CEA + cells was higher in patients with stage II–III and IV than in patients with stage I. A lower number of these cells indicated a higher 3-year RFS.	NA
	Kuroda et al.^380^	prognostic value	Ficoll	EpCAM, FGFR2, CD45	100 GC	FGFR2 + CTCs (≥5 cells/10 mL blood) showed poorer RFS.	NA
	Nevisi et al.^244^	prognostic value	CellSearch	EpCAM, HER2	105 GC	HER2-expression on CTCs was an independent prognostic factor for both overall and progression-free survival.	NA
Colorectal cancer	Wang et al.^381^	prognostic value	magnetic isolation	chromosome enumeration probe	130 CRC with stage II-III	Postoperative CTCs were significantly correlated with poor RFS.	NA
	Bidard et al.^382^	prognostic value	CellSearch	EpCAM, CK	153 CRC with liver metastasis	Baseline CTCs ≥3 was detected in 19% of the patients. CTC ≥ 3 at baseline and 4 weeks after therapy showed shorter OS.	NCT01442935
	Wang et al.^383^	prognostic value	Cyttel	Chromosomes 8 and 17 H1	121 CRC	CTCs were detected in 58.7% of CRC patients. Advanced CRC patients with CTC-positive had worse PFS and OS.	NA
	Messaritaki et al.^384^	prognostic value	Density gradient isolation, RT-qPCR	CEACAM5, EpCAM	198 advanced CRC	CEACAM5 was a dynamic adverse prognostic CTC biomarker in patients with metastatic CRC.	NA
	Su et al.^252^	prognostic value	CellSearch	EpCAM, CK, vimentin, twist, PRL-3	156 CRC	CTCs were detected in 100% of CRC patients. The count of mesenchymal and PRL-3+ CTCs ≥ 12 was significantly associated with recurrence and shorter DFS.	NA
	Sastre et al.^245^	prognostic value	CellSearch	EpCAM, CK	1202 metastatic CRC	Baseline CTCs ≥3 was detected in 41% of the patients.	NCT01640405, NCT01640444
	Pan et al.^385^	prognostic value	magnetic isolation	CK19	149 CRC	CTC counts were associated with TNM stages. The change escalated more rapidly in the CTC-positive group.	NA

*AR* androgen receptor, *BC* breast cancer, *CK* cytokeratin, *CTC* circulating tumor cell, *CRC* colorectal cancer, *DAPI* 4’,6-diamidino-2-phenylindole, *DFS* disease free survival, *DRFS* distant recurrence-free survival, *EMT* epithelial-mesenchymal transition, *GC* gastric cancer, *HCC* hepatocellular carcinoma, *HER2* human epidermal growth factor receptor-2, *mBC* metastatic breast cancer, *mCRPC* metastatic castration-resistant prostate cancer, *NA* not applicable, *NSCLC* non-small-cell lung cancer, *OS* overall survival, *PC* prostate cancer, *PDAC* pancreatic ductal adenocarcinoma, *PD-L1* programmed cell death ligand-1, *PFS* progression free survival, *qRT-PCR* quantitative real time polymerase chain reaction, *RCC* renal cell carcinoma, *RFS* recurrence free survival

### Evaluation of the cancer prognosis

The prognostic value of CTCs has been extensively studied. CellSearch is the only FDA-approved system for CTC detection used clinically. Based on CellSearch system^46,232–245^, CTCs represent an independent prognostic factor. Studies evaluating other CTC detection systems such as CanPatol, CTC-chip obtained the similar results. CTC enumeration is the main target of investigation, with a cut-off value of ≥5 for positivity, which usually indicates a worse prognosis. It is generally considered that increased CTC counts are correlated with higher likelihood of metastasis and cancer aggressiveness. In a meta-analysis pooling 2239 breast cancer patients including 21 studies^246^, the CTC count before neoadjuvant chemotherapy had a detrimental and decremental impact on patient survival, and patients with one, two, three to four, and five or more CTCs displayed a HR of death (95% CI) of 1.09 (0.65–1.69), 2.63 (1.42–4.54), 3.83 (2.08–6.66), and 6.25 (4.34–9.09), respectively. Furthermore, elevated baseline CTCs levels were associated with inferior survival, the presence of CTC clusters often predicted poor prognosis^247–249^, and increasing CTC counts or failure to clear CTCs during treatment was also a prognostic factor for worse survival^234,250,251^. Many studies have found that the molecular phenotypes of CTCs have strong prognostic value. EMT and stemness are the main molecular phenotypes of CTCs studied clinically. CTCs with expression of mesenchymal^252^ or stemness^253^-related markers associated with inferior survival. The expression of other molecular markers, such as HER2^46^, CD47^254^, PD-L1^254^ also have prognostic implications. Most studies investigate the prognostic value of CTCs at a single timepoint, while intriguingly, some studies have taken CTC dynamics into account. Magbanua et al.^237^ developed a novel latent mixture model to stratify groups with similar CTC trajectory patterns during the treatment course, and they found that analysis of serial CTCs can further stratify the patients of poor prognosis into distinct prognostic subgroups. The dynamic changes of CTCs may act as a surrogate prognosis biomarker over the long course of cancer progression. Given the rapid advancements in the accessibility and strengthening of sequencing technologies at the single-cell level, we may expect that in the future, genomic/transcriptional profiles of CTCs may serve as an outstanding prognostic marker, representing biological information that is more comprehensive and more closely related to prognosis. Studies of CTCs for predicting prognosis in recent three years are summarized in Table 3.

### Monitoring of the therapeutic response

In many clinical trials, CTCs have been used as useful biomarkers for monitoring cancer treatment responses^234,255–257^, either combined with imaging examinations, serum biomarkers or alone. Researchers prefer to involve CTCs in the evaluation of therapeutic efficiency, in view of the higher sensitivity of CTCs than imaging examination in some cases^9^. As a non-invasive method, the detection of CTCs may also contribute to avoiding frequent radiation exposure from imaging studies during the evaluation of treatment response. Most studies found a decrease or clearance in CTC counts was associated with a good therapeutic response, while the increase of CTC counts signified the opposite^258,259^. The Response Evaluation Criteria in Solid Tumors (RECIST) guidelines are the most often used standard for evaluating therapeutic response in solid tumors. However, in some studies, changes in CTC following therapy were not correlated with RECIST responses in cancer patients^260^. Indeed, CTCs assessment has not been included in the RECIST guidelines. Some CTCs measurement technologies have been recently developed to achieve genotyping for CTCs, which can also detect crucial gene mutations, such as ER^39,49^, HER2^39,49^, EGFR^261^, KRAS^262^, and TP53^263^, thus helping clinicians in treatment personalization and resistance options at the time of tumor progression. Studies using CTCs to monitor treatment response in recent three years are summarized in Table 3.

The great potential for CTCs in the clinical application of cancer diagnostics has emerged, although clinically, its use as a surrogate biomarker for cancer screening, treatment monitoring, and prognosis predicting is still limited. Once metastasis occurs, repeat biopsies of metastatic lesions are usually difficult to obtain, and different metastases are heterogenous even in the same patients. CTCs testing using peripheral blood samples is convenient, and may be more representative of the traits of metastatic cells, which are derived from different metastatic lesions in patients. Nevertheless, there is still a lack of guidelines for the clinical use of CTCs, such as a standardized CTCs detecting assay for different cancers, a combination diagnostic scheme with other clinical examinations, and indications for the appropriate timepoints for blood sampling.

## Discussion

Studies investigating CTCs have the great potential to reveal the fundamental processes of metastases, including the mechanisms involved in extravasation of CTCs from the primary tumor, how CTCs interact with blood cells to survive in the circulatory microenvironment, and how CTCs intravasate into the distant metastatic site to initiate new lesions. Significant molecular traits of CTCs can greatly contribute to identify targets for anti-metastatic therapies. Only a small proportion of CTCs can finally generate metastases, thus studies focusing on these strongly metastatic CTCs may provide deeper insights into CTCs-related therapeutic targets.

Various CTCs detecting technologies have emerged, however, the sensitivity and specificity of these technologies still need to be further improved. Epithelial marker-based CTC detection technology, such as the CellSearch system has opened a new era for CTC analysis and clinical applications, but their drawbacks are rapidly being acknowledged and appreciated by researchers. EMT is a crucial trait of metastatic cancer cells, indicating insufficient capture efficiency of epithelial marker-based CTC detection technology. However, mesenchymal marker-based detection technologies may also be contaminated by non-CTCs, such as tumor-associated fibroblasts and endothelial clusters, which induce the risk of false positivity. Nevertheless, it is intriguing that recent studies have reported that those non-cancerous tumor-derived cells presented in cancer patients are also important surrogate biomarkers for cancer patients^264^. Cancer-type-specific molecular markers for CTCs are likely another option, as CTCs of different cancer types possess different molecular markers. However, the sensitivity and specificity of known cancer-type-specific CTC markers are not satisfactory. Physical-property-based CTC detection technologies also have the problem of contamination by non-CTCs, especially for those with similar physical properties as CTCs. Microfluidic-based and nanotechnology-based CTC detection technologies have become popular in recent years, while the efficiency of these technologies still needs further large-scale clinical validation. High cell detection efficiency and contamination removal capability are the two key strengths of a successful CTC detection technology, while substantial technical optimization of CTC detection is urgently needed to achieve these requirements.

Comprehensive characterization of CTCs is lacking. The limited amount of genomic DNA, RNA, and protein content of CTCs is a bottleneck for exploring their genome, transcriptome, epigenome, and proteome properties. Nonetheless, the emerging genome and transcriptome studies of CTCs have recently profited from the fast-evolving technology of single-cell sequencing, while the proteome studies of CTCs are still elusive due to the very limited technologies for proteome exploring at a single-cell level. However, the study of the CTC proteome is imminent, not only because it can provide a picture of the biological characterization of CTCs, but also because it can help to discover CTC-specific membrane proteins which may help optimize CTC detection.

As for the solid tumor microenvironment, the blood microenvironment around CTCs also plays a significant role in tumor survival and invasion capacity. However, knowledge of the underlying mechanisms behind the survival of CTCs is still limited, as it is a complex process that involves not only shear forces and fluid mechanics but also soluble factors and tumor-associated extracellular vesicles, which are not detailed here. Furthermore, it remains to be confirmed whether CTC clusters are more suitable for interacting with other blood components or adapting to shear forces than single migratory CTCs. If combined with specific biomarkers for strategically detecting CTCs and the interaction of CTCs with associated peripheral blood cells, we could improve the clinical practicability and monitoring power of CTCs by obtaining more comprehensive information on tumor burden and immune status of patients.

Although CTCs have shown initial promise in clinical applications, many challenges must still be overcome before CTC analysis can be widely applied in the clinic. Today, the clinical application of CTCs mainly depends on the analysis of CTC cell enumeration and molecular phenotypes. A more comprehensive characterization of CTCs based on their genome, transcriptome, and proteome with high-throughput sequencing will further benefit clinical application, but also add to the complexity and difficulty of data analysis. CTCs will be a crucial component of “Precision medicine” in the future, as the phenotypic, genotypic, and functional characterization can provide an opportunity to study drug susceptibility that is related to metastasis. The genome and transcriptome analysis of CTCs can unveil potential drug targets. Viable CTCs for drug sensitivity/resistance testing over the therapy course can guide precision medication. However, the culture of CTCs is very challenging: (1) limited methods are available to isolate viable CTCs, which also yield low numbers of CTCs and (2) a favorable circulatory microenvironment for CTC survival is difficult to mimic. Very limited CTC-derived cell lines from cancer patients have been established. Optimization of CTC culture conditions will be needed.

Furthermore, CTCs, circulating tumor DNA (ctDNA), and exosomes are all present in liquid biopsy samples. An exploration of the advantages and disadvantages of each substrate present in the liquid biopsies, and how to better incorporate them into clinical application is needed to achieve more precise diagnoses. Among liquid biopsy methods, CTCs have tremendous advantages, as isolated CTCs can be viable, which can optimize CTC-derived explants or three-dimensional organoid cultures for functional testing or for drug-screening assays. The study of CTCs is attractive, and CTC detection may likely become an essential component of cancer management in the future. As the picture becomes clearer, we are fully confident about the promising potentials of CTCs.
